# Magnitude and Risk Factors for Paediatric Congenital Heart Surgery Complication and its association with Patient Outcomes in the Cardiac Center of Ethiopia

**DOI:** 10.4314/ejhs.v34i4.6

**Published:** 2024-07

**Authors:** Mohammed Nasir Beshir, Muluken Ahmed, Temesgen Tsega, Tadesse Getahun

**Affiliations:** 1 Pediatrician, Pediatrics Cardiologist, Hawassa University, Hawassa; 2 Pediatrician, Pediatric Cardiology Fellow, Arba Minch University, Arba Minch; 3 Tsega Pediatrician, Pediatrics Cardiologist, St Paul's Millennium Hospital Medical College, Addis Ababa; 4 Pediatrician, Pediatrics Cardiologist, Yekatit 12 Hospital Medical College, Addis Ababa

**Keywords:** Congenital heart diseases, surgery, complications, outcome

## Abstract

**Background:**

Congenital heart disease surgery is related to significant postoperative complications which have been associated with poor patient outcomes. However, the prevalence, predictors, and effect of complications on outcomes have only been evaluated in a few numbers of studies. This study aimed to assess the magnitude of postoperative complications after congenital heart surgery, their predictors, and the associations between complications and patient outcomes.

**Methods:**

A retrospective, single-center cross-sectional study was done at the Cardiac Center of Ethiopia (CCE) on children who had undergone cardiac surgery between 2009 and 2022. All 919 pediatric patients aged below 18 years who had undergone congenital heart surgery were included in the study.

**Result:**

Of the 919 patients who underwent surgery in the cardiac center of Ethiopia, Ventricular septal defect (VSD) was the most common diagnosis (41%) and VSD patch closure (39.4%) was the most common surgical procedure. The presence of at least one complication was identified in 39.5% of patients. Of all patients, 11.3% had cardiac, 23.1% had extra-cardiac complications, and 5.3%% had major complications. Higher age at diagnosis, lower weight at admission, cyanotic congenital heart disease, higher Risk adjustment for congenital heart surgery score (RACH-1 score), higher vasoactive inotropic score, Cardiopulmonary bypass use during surgery, higher cardiopulmonary bypass time, higher aortic cross-clamp time, higher duration of surgery, and the higher number of surgeries were associated with complications. The duration of mechanical ventilation, length of intensive care unit stays, and length of hospital stay was significantly prolonged in patients with complications.

**Conclusion:**

Congenital heart disease surgeries pose a high risk of complications, and these complications are associated with poor patient outcomes in Ethiopia. Therefore, predicting complications based on risk factors and early detection and treatment is crucial to improve the patient's outcome.

## Introduction

Congenital heart disease (CHD) prevalence was 8 per 1000 live births in 2017 and is on the rise (1). It makes up one-third of all major congenital anomalies(2). It has multiple complications like heart failure, endocarditis, arrhythmias, pulmonary hypertension, and death(3). Therefore, the likelihood of patients surviving into adulthood depends on having access to the best possible care, such as timely surgery (4).

However, CHD surgery itself has a risk of mortality and complications in several organs (4). The reported perioperative complication (POC) rate reaches up to 40%(5). Fortunately, postoperative morbidity and mortality have dramatically decreased as a result of improvements in perioperative care, advanced surgical management options, and imaging modalities that facilitate early diagnosis and management of complications (6).

Patient safety is given more emphasis nowadays; therefore, complication reporting is crucial for quality improvement and indicating the performance of a service (6,7). Additionally, the occurrence of complications may have an impact on the patient's cost, mortality rate, intensive care unit (ICU) stay, and duration of mechanical ventilation; hence, reporting complications with wise anticipation after identifying a set of predictors is essential (8).

Complication patterns and prevalence demonstrated significant variation across centers. Moreover, there are just a few studies that evaluate the effect of complications on hospital stay, intensive care unit (ICU) stay, stay on mechanical ventilation, and mortality. Hence, the purpose of this study is to evaluate CHD surgery complications and their predictors, as well as the effect of complications on mortality, length of hospital stays, stays in the intensive care unit, and stays on mechanical ventilation in patients who underwent surgery in the cardiac center of Ethiopia.

## Methods and Materials

A cross-sectional study was conducted on patients who underwent congenital heart surgery between January 2009 and January 2022 at the Cardiac Center of Ethiopia. All 919 pediatric congenital heart disease patients aged below 18 years of age who had undergone heart surgery in the Cardiac Center of Ethiopia were included in this study. The Cardiac Center of Ethiopia is located in Addis Ababa, Ethiopia's capital city. Currently, the center has five surgeons, five cardiologists, two operating rooms, and one intensive care unit with 10 beds. It has two GE 9, one Samsung, and one vivid echocardiography for preoperative, intraoperative, and postoperative patient evaluation. There is one transesophageal echocardiography (TEE), which is utilized during surgery when required. The Center is mostly supported by donations, and the services are free. Two to three surgeries are performed each week. Before 2017, surgeries were performed by foreign surgeons; however, as of 2017, local teams have taken over the majority of the surgical service delivery.

The dependent variables of this study were complications of congenital heart surgery, outcome measures (total length of mechanical ventilation (TLMV), total length of ICU stays (TLICUS), total length of hospital stays (TLHS), Mortality of congenital heart surgery, and Reoperation for congenital heart surgery). Independent variables were socio-demographic characteristics (age at diagnosis, age at surgery, sex, weight, and height), clinical characteristics (type of CHD, Preoperative risk factors, New York Heart Association (NYHA)/Rose class), and perioperative characteristics (risk adjustment for congenital heart disease (RACH-1) category, Vasoactive inotropic score (VIS), cardiopulmonary bypass time (CBP use), Nadir temperature during CBP, Nadir Hct during CBP, Nadir temperature during CBP, Heparin dose during CBP, CPB time, aortic clamp time (AoX time), reperfusion time, duration of surgery, Residual shunt or obstructive lesion, Number of procedures).

**Data collection procedure and tools**: A structured questionnaire was used for data extraction. We extracted the data from the operation room log book and patient medical records. The questionnaire had three parts that assessed the socio-demographic characteristics, clinical characteristics, and perioperative characteristics of the patient. ICD-10 was used to screen for the primary diagnosis of CHD(9). Preoperative risk factors, surgical procedures, and postoperative complications were identified and categorized by the Society of Thoracic Surgeons Congenital Heart Surgery Database data collection form (version 3.0) (10).

**Data quality assurance**: To ensure the internal validity of the study, the maximum effort was taken to assure the quality of the data and minimize errors and bias using the following measures: two days of training for data collectors and supervisors were carried out to have a clear understanding of the objective of the study and the data collection procedure. The pre-testing of the structured format was conducted on 10% of the sample at the cardiac center in Ethiopia before the data collection process, and the format was checked for completeness daily by immediate supervisors and the principal investigator. After checking for consistency and completeness, the supervisors submitted the filled-out questionnaire to the principal investigator, who also rechecked. Data were cleaned and entered by the principal investigator, and strict daily supervision and spot-checking were carried out.

**Data analysis**: The data were checked manually for completeness. Then the data were cleaned and stored for Consistency after being entered into Epi Info 7 software. For further analysis, the data were re-exported to SPSS version 27.0 software and Microsoft Excel 13. POCs were divided into cardiac and extra-cardiac types. Patient characteristics and outcomes were summarized and separated into two groups based on the presence of the POCs. Continuous variables after checking for normality are summarized by the median and interquartile range (IQR). Categorical variables were summarized by absolute frequency and percentages. Tables and graphs were used to present the data. Binary logistic regression was used to determine the association between POCs and socio-demographic, clinical, and perioperative factors. The odds ratio was used to assess the strength of the association. Median regression was used to assess the difference in medians in the length of duration on mechanical ventilation (LOMV), length of ICU stays (LICUS), and length of hospital stays (LHS) between patients with cardiac, extra-cardiac, or any POCs and patients with cardiac, extra-cardiac, or any POCs. An odds ratio with a 95% CI was used to demonstrate the median difference. Fisher's exact test was used to assess the association of cardiac, extra-cardiac, or any POCs with operative mortality and reoperations. A p-value of <0.05 was considered statistically significant in all analyses in this study.

**Operational definitions and study outcomes**: The complication in this study was defined as an event or occurrence associated with surgery that departs from the expected course of events. The postoperative complication is any complication that arises between the operating room entry date and time and the end of the period of surgical data collection, which encompasses complications that occur intra-operatively and postoperatively. Patients who had one of these listed complications: low cardiac output, complete or transient heart block requiring a permanent or temporary pacemaker, mild, moderate, or severe left ventricular dysfunction, hypertension, arrhythmia other than heart block requiring intervention, or superior vena cava stenosis, were categorized as having a cardiac complication.

Patients who had one of these complications: acute kidney injury, pulmonary hypertension or pulmonary hypertension crisis, pleural effusion or chylothorax, pneumothorax, atelectasis, haemothorax, pneumonia, laryngeal nerve palsy, diaphragmatic palsy, hyperglycemia needing intervention, metabolic acidosis patients with hyperkalemia needing intervention, metabolic alkalosis with hypokalaemia needing intervention, hypocalcemia needing intervention, and hypoglycemia, were labelled as having extra-cardiac complications. The focal neurologic deficit, seizure, bleeding needing a transfusion, sepsis with multi-organ failure, and wound infections are also labelled as extra-cardiac complications.

Major complications were defined as having one or more of the following complications: renal failure requiring dialysis, permanent neurologic deficit, pacemaker, paralyzed diaphragm, mechanical circulatory support, and unplanned reintervention.

We used echocardiographic grading for pericardial effusion and pleural effusion. The echocardiographic grading of pericardial effusion and pleural effusion we used was echo-free space between the heart and pericardium or echo-free space between the diaphragm and lung. Pericardial effusion grading is made by measuring echo-free space during diastole, and space less than 10 mm is considered mild; space between 10 and 20 mm is considered moderate; and space beyond 20 mm is considered large, respectively. Left ventricular dysfunction grading: mild dysfunction as left ventricular ejection fraction (LVEF) between 40% and 49%, moderate dysfunction between 30% to 39%, and severe dysfunction less than 30%, were used in this study.

Superior vena cava (SVC) stenosis was defined in this study as flow acceleration in the SVC with a peak Doppler gradient above 3cm/s. Acute kidney injury was defined as an absolute increase in serum creatinine by ≥0.3 mg/dL or an increase in serum creatinine ≥1.5x above baseline. Pulmonary hypertension is diagnosed a by peak tricuspid regurgitation (TR) gradient above 2.8cm/s. Chylothorax was defined by the passage of milky pleural fluid with high lymphocyte and triglyceride during pleural tap. Paradoxical abdominal movement clinically, elevated hemidiaphragm on chest X-rays, and paradoxical movement of the diaphragm in echocardiography were used to diagnose diaphragmatic palsy. The laryngeal nerve palsy was diagnosed clinically if there was a voice change after surgery.

Metabolic complications were diagnosed by arterial blood gas analysis (ABG) by our laboratory reference range. Hyperglycemia was diagnosed by a random blood sugar level above 180 mg/dl, and hypoglycemia by a random sugar level below 50 mg/dl. Operative mortality was defined as any death, regardless of cause, that (1) occurs within 30 days of surgery, in or out of the hospital, and (2) occurs after 30 days when the patient is still being treated at the same hospital after the operation. Reoperation was defined in this study as repeated surgery that is necessary within 30 days after the initial surgery because of complications.

The primary outcome in this study was the presence of any one of the aforementioned complications and their patterns in the perioperative period. Our secondary outcome was the effect of complications on established outcomes (length of stay in mechanical ventilation, length of ICU stays, length of hospital stays, operative mortality, and need for reoperation).

**Ethical consideration**: Ethical clearance was obtained from the ethical review committee and Advisor of Saint Paul Millennium Medical College before the actual research work was conducted. The objective of the study was explained to the medical director of CCE, and consent was obtained to extract data from patient charts. To assure confidentiality, the data were extracted without any personal identifiers and used only for this research purpose.

## Results

Socio-demographic and clinical characteristics: Table 1 presents the socio-demographic and clinical characteristics of patients separated by the presence of complications. As demonstrated in the table, patients' median ages at diagnosis and surgery were 2 years (IQR: 2–3 years) and 7 years (IQR: 3– 13 years), respectively, with a 4:1 ratio of female to male patients.

**Table 1:** The sociodemographic and clinical characteristics of patients who have undergone surgery in the Cardiac Center of Ethiopia separated by complication occurrence, 2009-2022

Variable	All patients (n=919)	Postoperative complications				

		No	Yes	COR (95%CI)	AOR (95%CI	*P value*
Age at diagnosis in years, median (IQR)	2(2-3)	2(2-12)	2(1-2)	0.76(0.71-0.81)	0.83(0.76-0.9)	<.001
Age at the surgery in years, median (IQR)	7(3-13)	13(13-14)	6(3-13)	0.8(0.77-0.83)	0.92(0.75-1.9)	.70
Gender						
Female, n (%)	736(80.1)	485(87.2)	251(69.1)	1	1	
Male, n (%)	183(43.7)	71(12.8)	112(30.9)	3(2.2-4.3)	0.77(0.44-1-4)	.40
Weight in Kg, median (IQR)	40(19-40)	40(40-41)	20(11-38)	0.92(0.91-0.93)	0.95(0.88-0.95)	.03
Height in cm, median (IQR)	145(112-148)	145(145-148)	112(90-140)	0.96(0.95-0.97)	0.98(0.9-1.3)	.50
Types of CHD						
Acyanotic, n (%)	995(94.5)	549((52.1)	446(42.4)	1	1	
Cyanotic, n (%)	58(5.5)	7(0.7)	51(4.8)	8.4(3.7-19.1)	1.1(1.01-2.85)	.04
Presence of preoperative risk factor Yes, n (%)	30(3.3)	10(1.8)	20(5.5)	3.2(1.5-6.9)	2.6(0.9-7)	.06
NYHA/ Rose class at admission						
Class 1 or 2, n (%)	700(76.3)	469(51.1)	231(25.2)	1	1	
Class 3 or 4, n (%)	219(23.9)	87((9.5)	132(14.4)	3(2.3-4.2)	0.7(0.35-1.2)	.16

**Figure 1:**
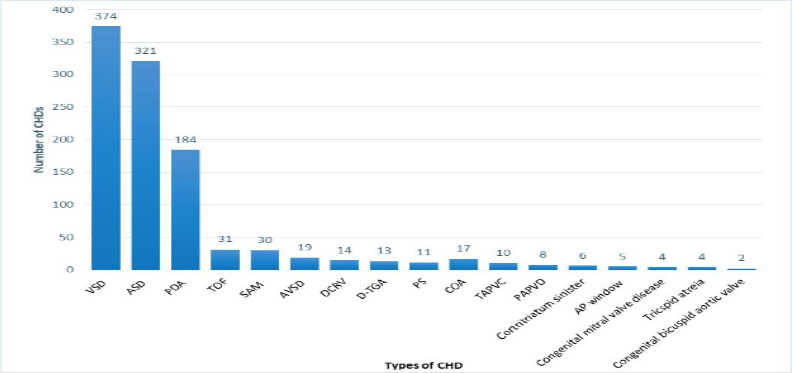
The types of CHDs and their magnitude in patients who have undergone surgical intervention at the Cardiac Center of Ethiopia- VSD: Ventricular septal defect; ASD: Atrial septal defect; PDA: Patent ductus arteriosus; TOF: Tetralogy of Fallot; SAM: subaortic membrane; AVSD: Atrioventricular septal defect; DCRV: Double chamber right ventricle; D-TGA: D-Transposition of great arteries; PS: Pulmonary stenosis; COA: Coarctation of aorta; TAPVC: Total anomalous pulmonary venous connection; PAPVD: Partial anomalous venous drainage; AP window Aortopulmonary window

More than 90% of patients had acyanotic CHDs. The first three most common CHDs were VSD (41%), ASD (35%), and PDA (20%). More than 75% of the patients were in NYHA/Rose class 1 and 2 heart failure categories at admission. Preoperative risk factors were present in only 30 (3.3%) patients, of whom 12 (1.3%) had Down syndrome, 6 (0.7%) had congenital rubella syndrome, 3 (0.3%) had scoliosis, 3 (0.3%) had old vegetation, 2 (0.2%) had Holt-Oram syndrome, 2 (0.2%) had Turner syndrome, and 2 (0.2%) had Marfan syndrome (Figure 1).

**Figure 2:**
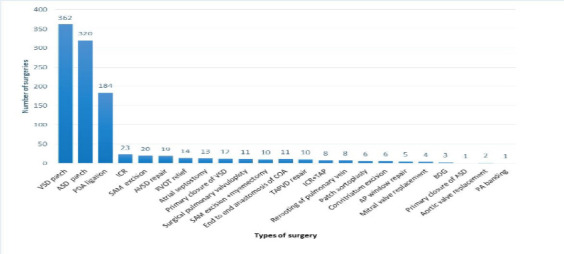
The types of surgery and their spectrum in patients who have undergone surgical intervention at the Cardiac Center of Ethiopia- VSD patch: Ventricular septal defect patch closure; ASD patch: Atrial septal defect patch closure; PDA ligation: Patent ductus arteriosus ligation; ICR: Intracardiac repair of Tetralogy of Fallot; SAM excision: subaortic membrane excision; AVSD repair: Atrioventricular septal defect repair; RVOT relief: Right Ventricular outflow tract muscle resection; Primary closure of VSD: Primary closure of Ventricular septal defect; End to end to the anastomosis of COA: End to the anastomosis of Coarctation of the aorta; TAPVC repair: Total anomalous pulmonary venous connection repair; AP window: Aortopulmonary window repair; BDG: Bidirectional Glenn: Primary closure of ASD: Primary closure of atrial septal defect; PA banding: Pulmonary artery banding

**Figure 3:**
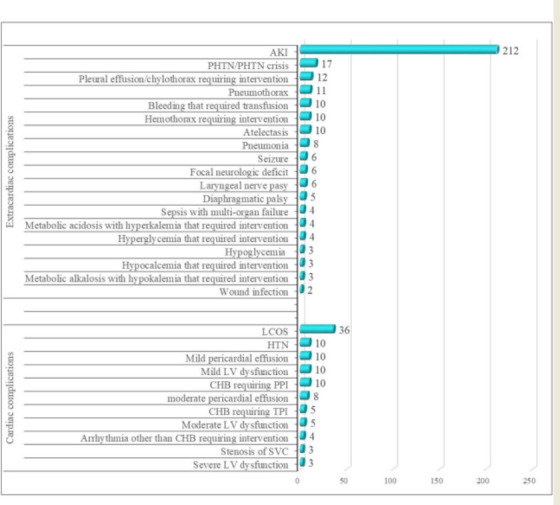
Patterns of cardiac and extracardiac complications- PHTN: Pulmonary hypertension; AKI: Acute kidney injury; SVC: Superior vena cava; LV: left ventricle; CHB: Complete heart block; TPI: Transient pacemaker implantation; PPI: Permanent pacemaker implantation; LCOS: Low cardiac output syndrome

Figure 2 summarizes the type and patterns of 1053 congenital heart surgeries done in 919 patients. The three most common surgeries were VSD patch closure at 39.4%, ASD patch closure at 30.4%, and PDA ligation at 20%. Of the 919 patients, 363 (39.5 % of them) had at least one complication associated with surgery (Figure 3).

Cardiac complications occurred in 104 (11.3%) patients. Low cardiac output syndrome (LCOUS), complete heart block (CHB) requiring permanent or temporary pacemaker implantation, arrhythmia other than heart block requiring intervention, mild, moderate, and severe left ventricular dysfunction, mild pericardial effusion, hypertension, and superior vena cava stenosis were the cardiac complications identified.

Of all, 336(36.6%) patients experienced extracardiac complications; 212 (23.1%) had renal complications (the only identified renal complication was acute kidney injury(AKI) and 10(1.1%)) patients need dialysis), 79(8.6%) patients had pulmonary complications (the identified pulmonary complications were pulmonary hypertension, pulmonary hypertension crisis, pleural effusion, chylothorax, pneumothorax, atelectasis, hemothorax, pneumonia, laryngeal nerve palsy, and diaphragmatic palsy), 17(1.8%) had metabolic complication (that included hyperglycaemia needing intervention, metabolic acidosis patients with hyperkalaemia needing intervention, metabolic alkalosis with hypokalaemia needing intervention, hypocalcaemia needing intervention, and hypoglycaemia), 12(1.3%) had neurologic complications (that included focal neurologic deficit, seizure), 10(1.1%) patients had hematologic complications( bleeding that required transfusion was the only hematologic complication), 4(0.4%) patients had sepsis with multi-organ dysfunction, and wound infection occurred in 2(0.2%) patients. Major complications occurred in 49 (5.3%) patients: 4 reoperations, 12 focal neurological deficits, 10 pacemaker requirements, 10 needs for dialysis, and 5 diaphragmatic palsies.

The association of sociodemographic, clinical, and perioperative factors with complications was assessed, and the results are depicted in Tables 1 and 2. As seen in the tables, each additional increase in age at diagnosis in one year and weight in one kg at admission was associated with a 17% and a 5% decrease in the odds of having complications after CHD surgery, respectively. The odds of complications increased by 10% in patients who had cyanotic CHDs compared to acyanotic ones. Patients who had undergone more than one surgery were 40% more likely to develop complications than patients who had undergone a single surgery. Patients in the RACH-3-RACH-4 category had odds of complications that were 3.5 times higher than those in the RACH-1 and 2 categories. The odds of complication increased by 10% in patients who underwent surgery using CBP compared to patients without CBP. An additional 1-minute increase in CBP and AoX time increased the odds of complications by 10% and 20%, respectively. There was a 20% higher likelihood of experiencing complications for every extra hour of the duration of surgery. A 30% higher likelihood of developing complications was observed for each additional unit of the VIS score (Tables 1 and 2).

**Table 1:** The perioperative characteristics of patients who underwent a Cardiac center in Ethiopia between 2009-2022

Characteristics	All patients (n=919)	Postoperative complications				

		No	Yes	COR (95%CI)	AOR (95%CI)	*P value*
RACH-1 category, n (%)						
RACH-1 and RACH -2, n (%)	862(94)	548(59.8)	314(34.2)	1	1	
RACH-3 and RACH -4, n (%)	57(6.2)	8(0.9)	49(5.3)	10.7(5-22.9)	3.5(1.2-9.6)	0.02
VIS, median (IQR)	7(3-15)	6(3-7)	15(6-20)	1.25(1.21-1.28)	1.3(1.24-1.37)	<.001
CBP, n (%)						
No	44(4.8)	29(3.2)	15(1.6)	1	1	
Yes	875(95)	527(57)	348(38)	1.2(0.31-1.86)	1.1(1.01-1.9)	.04
Nadir temperature in °c, median (IQR)	32(32-33)	32(32-33)	32(32-33)	1.7(1.32-2.2)	1(0.8-1.2)	.80
Nadir haematocrit during CBP in %, median (IQR)	30(28-32)	29(28-31)	29(28-31)	1(0.92-1.1)	1.1(0.9-1.2)	.50
Nadir temperature post CBP in ° c, median (IQR)	30(28-32)	34.9(34.4-35.6)	35.1(34.4-35.7)	1.2(0.94-1.4)	1.1(0.7-1.4)	.70
Heparin dose during CBP in IU/kg, median (IQR)	510(450-570)	510(450-570))	510(450-568)	0.99(0.98-1.1)	1(0.96-1.8)	.20
CBP time in min, median (IQR)	50(50-80)	50(50-51)	70(50-110)	1.03(1.03-1.04)	1.1(1.05-1.8)	.04
ACC time in min, median (IQR)	30(14-30)	19(13-30)	30(17-46)	1.03(1.02-1.04)	1.2(1.1-1.3)	<.001
Reperfusion time in min, median (IQR)	28(10-32)	12(10-27)	32(10-32)	1.05(1.04-1.07)	1(0.99-1.02)	.90
Duration of surgery in hrs, median (IQR)	1.3(1-3)	1.3(1-3)	2(1-2.9)	1.3(1.2-1.4)	1.2(1.1-1.3)	.04
Residual shunt or obstructive lesion n (%)						
No n (%)	797(86.7)	524(57)	273(29.7)	1	1	
Yes n (%)	122(13.3)	32(3.5)	90(9.8)	5.4(3.5-8.3)	0.6(0.3-1.1	0.07
Number of procedures						
1	817(89)	526(57)	291(32)	1	1	
≥2	102(11)	30(3)	72(8)	3.2(2.1-4.9)	1.4(1.2-2.4)	.03

Abbreviations: AoX time: Aortic cross-clamp time; CBP: cardiopulmonary bypass time IQR: Interquartile range; n (%): number (percent); RACH-1: Risk adjustment for congenital heart disease-1; VIS: Vasoactive inotropic score

**Table 2:** The median difference between patients who had any complications, cardiac complications, or extracardiac complications with patients who had no complications, in patients who undergone surgery in the Cardiac Center of Ethiopia, 2009-2022

Outcome measures	All Complications			

	No, the median (IQR)	Yes, the median (IQR)	Median difference (95%CI)	*P value*
TLMV, hrs.	2(2-2.1)	6(6-12)	4(3.7-4.3)	<.001
TLIS, days	3(3-3.1)	5(4-7)	2((1.9-2.1)	<.001
TLHS, days	4(4-4.1)	8(7-12)	4(3.8-4.2)	<.001
	**Cardiac complications**			
	No, the median (IQR)	Yes, the median (IQR)	Median difference (95%CI)	*P value*
TLMV, hrs.	3(3-5)	6(4-9)	3(2.9-5.1)	<.001
TLICU, days	3(3-5)	6(4-9)	3(2.8-5.2)	<.001
TLHS, days	4(4-7)	11(7-21)	7(6.7-7.3)	<.001
	**Extracardiac complications**			
	No, the median (IQR)	Yes, the median (IQR)	Median difference (95%CI)	*P value*
TLMV, hrs.	2(2-4)	6(6-12)	4(3.8-4.2)	<.001
TLICU, days	3(3-3.1)	5(3-7)	2(1.9-3.3)	<.001
TLHS, days	4(4-4.1)	8(6-12)	4(3.9-4.1)	<.001
	**Major complications**			
	No, the median (IQR)	Yes, the median (IQR))	Median difference (95%CI)	
TLMV, hrs.	2(2-6)	5(3-6)	3(1.5-4.5)	<.001
TLICU, days	3(3-5)	5(4-7)	2(1.1-4)	0.01
TLHS, days	4(4-7)	6(5-11)	2(1-3.9)	0.01

Abbreviations: TLHS: Total length of hospital stay; TLICU: Total length of intensive care unit stay; TLMV: Total length of mechanical ventilation

**Association of outcome with the occurrence of complications**: The median values of total length of mechanical ventilation in hours, total length of ICU stays in days, and total length of hospital stays in days between patients who had any complications (cardiac, extracardiac, or major) and patients with no complications were statistically different (Table 3). Twelve (1.3%) patients underwent reoperation for their complications, and 5 (0.9%) of those who underwent reoperation had complications, but 7 (1.9%) had no complications. However, unplanned reoperations were not associated with complications. Of all patients, seven (0.8%) died in the hospital, of whom four had reoperations. The presence of complications was associated with death (p<.001), and all the patients who died had at least one complication.

## Discussion

Compared to most other studies in the same area, our study showed more patients had complications, including cardiac and extra-cardiac. In addition, numerous factors associated with complications were identified. Patients with complications had a longer duration of ventilator use, longer ICU stays, and longer hospital stays, so there was a significant association between the presence of complications and outcome measures. This study showed a low mortality rate of 0.8%, which is consistent with recent estimates of a mortality rate of less than 1.64%(11). Similarly, the percentage of reoperations was relatively low.

The percentage of patients who suffered at least one complication in this study (39.5%) was higher than in studies conducted in Argentina and Brazil (8.3%, and 19.7% respectively)(12,13). whereas Indonesian and Iranian studies found 84.1% and 43.3% percentages of complications, which were lower in our study(14,15). Differences in the complexity of surgical procedures performed by centers and the use of different complication lists in data extraction can be used as explanations for this variation.

We reported fewer cardiac complications (11.3%) compared to studies conducted in the USA (25%) and Iran (34.4%), but greater than the China study (6.8%)(8,14,16). The use of different operational definitions for these cardiac complications could account for the results varying magnitudes. A study from the United States included extracorporeal oxygenation, reoperation, and heart block; however, a study from China omitted arrhythmia from the list of cardiac complications.

Similar to our study, studies conducted in the USA, China, and Indonesia showed that extracardiac complications occurred more frequently than cardiac complications, with extra-cardiac vs. cardiac percentages of 37 versus 25, 27.6 versus 6, and 51 versus 33.1, respectively(8,15,16). Variations in the list of extracardiac complications among studies may be responsible for the variation in magnitude.

The proportion of major complications in our study is lower than in a multi-center study done in the USA and Argentina(12,17). This disparity can be explained by the variations in the case mix between facilities.

A study from China demonstrated that patients with complications are younger and lighter than their children who do not have complications, which is consistent with our findings(16) which can be explained by the increased risk of anesthesia intra-operatively and drug allergy in the perioperative period in young age.

The results of our study showed a relationship between complications and cyanotic congenital heart disease. This was supported by a study done in Indonesia that showed Cyanotic heart disease had more risk for complications(15). In these cases, studies carried out in China have shown that patients with reduced blood oxygen saturation are at increased risk of complications(16). The higher complication frequency in surgery for cyanotic heart disease is due to the longer duration of the surgery it takes, and the high risk of metabolic acidosis and hypoxemia in this group of patients.

In this study, Patients undergoing cardiac surgery with RACH-1 scores of 3 and 4 exhibited higher rates of complications which is consistent with one US study(8). This could be because procedures become more complex as the RACH-1 category score increases. In contrast to our data, Saudi research has found no statistically significant relationship with RACH-1 category (18).

In our study, VIS was found to be associated with complications following surgical procedures, in line with studies from the USA and Finland(19,20). Higher VIS indicates that there is poor hemodynamic status in the patient, which may compromise his or her surgical outcome.

CBP increases organ damage by stimulating inflammatory pathways, so there may be significant differences in the incidence of complications between patients who received CBP and those who did not (21). The systemic impact of CPB owing to the systemic inflammatory response and occurrence of multiple organ ischemia in aortic clamping may account for this. The study conducted in the USA, on the other hand, did not show any clear difference between CBP operations and those that had not been handled by CBP (8).

In addition to our study, studies conducted in China, Indonesia, and Egypt have shown that aortic cord clamping and cardiopulmonary time were associated with complications(15,16,22). The systemic impact of CPB owing to the systemic inflammatory response and the possible occurrence of multiple organ ischemia in aortic clamping may account for this.

Our study's findings, which are consistent with those conducted in China, indicate that complications following congenital heart disease surgery are predicted by longer surgery times(16). Additionally, our study has demonstrated that the likelihood of complications rises with the number of surgeries performed. It has been reported that many operations requiring prolonged operating times are more susceptible to surgical site infection, venous thromboembolism, bleeding, hematoma formation, and necrosis(23). This might have been brought on by the surgical team's increased levels of fatigue and the extended use of anesthetic. Similarly, as the number of surgeries might be associated with surgeon fatigue and concentration.

Our study showed a significant relationship between postoperative complications and mechanical ventilation duration, prolonged stays in intensive care units, and longer hospital stays or deaths based on studies carried out in the US, Argentina, China, and Saudi Arabia(8,12,16,18). The reason for these prolonged hospital stays is that patients with complications require more supportive care or surgical intervention.

In conclusion, congenital heart disease surgeries pose a high risk of complications and have been associated with negative patient outcomes. Therefore, prediction of complications based on a set of predictors, early detection, and treatment of complications are crucial to improve the patient's outcome.

## Limitations of the study

This study has a few limitations. First, because the study was retrospective, classification bias might have been more likely to occur. Second, if it was conducted in only one setting, generalizability may be challenging. Last but not least, it is challenging to associate a particular congenital heart disease operation with a specific consequence. However, this study may serve as a starting point for future research on particular congenital cardiac anomalies and assist in quality improvement by allowing for a comparison of the center's results to those of other centers.
